# Ginsenoside-Rd Promotes Neurite Outgrowth of PC12 Cells through MAPK/ERK- and PI3K/AKT-Dependent Pathways

**DOI:** 10.3390/ijms17020177

**Published:** 2016-01-29

**Authors:** Song-Di Wu, Feng Xia, Xue-Mei Lin, Kang-Li Duan, Fang Wang, Qing-Li Lu, Huan Cao, Yi-Hua Qian, Ming Shi

**Affiliations:** 1 Department of Human Anatomy, Histology and Embryology, School of Basic Medical Sciences, Xi’an Jiaotong University Health Science Center, 76 Yanta West Road, Xi’an 710061, China; wusongdi@gmail.com; 2 Department of Neurology, First Hospital of Xi’an, Xi’an 710002, China; mag_little@163.com (X.-M.L.); duankangli2016@sina.com (K.-L.D.); wjhy1234@163.com (F.W.); luql525@163.com (Q.-L.L.); neurocao@163.com (H.C.); 3 Department of Neurology, Xijing Hospital, the Fourth Military Medical University, Xi’an 710032, China; xiafeng@fmmu.edu.cn

**Keywords:** ginsenoside Rd, neurite outgrowth, ERK, AKT, GAP-43, PC12 cells

## Abstract

*Panax ginseng* is a famous herbal medicine widely used in Asia. Ginsenosides have been identified as the principle active ingredients for *Panax ginseng*’s biological activity, among which ginsenoside Rd (Rd) attracts extensive attention for its obvious neuroprotective activities. Here we investigated the effect of Rd on neurite outgrowth, a crucial process associated with neuronal repair. PC12 cells, which respond to nerve growth factor (NGF) and serve as a model for neuronal cells, were treated with different concentrations of Rd, and then their neurite outgrowth was evaluated. Our results showed that 10 μM Rd significantly increased the percentages of long neurite- and branching neurite-bearing cells, compared with respective controls. The length of the longest neurites and the total length of neurites in Rd-treated PC12 cells were much longer than that of respective controls. We also showed that Rd activated ERK1/2 and AKT but not PKC signalings, and inhibition of ERK1/2 by PD98059 or/and AKT by LY294002 effectively attenuated Rd-induced neurite outgrowth. Moreover, Rd upregulated the expression of GAP-43, a neuron-specific protein involved in neurite outgrowth, while PD98059 or/and LY294002 decreased Rd-induced increased GAP-43 expression. Taken together, our results provided the first evidence that Rd may promote the neurite outgrowth of PC12 cells by upregulating GAP-43 expression via ERK- and ARK-dependent signaling pathways.

## 1. Introduction

*Panax ginseng* is a traditional herbal medicine popular in China, Korea and Japan. A group of studies have demonstrated that ginseng has a wide range of beneficial effects in the treatment of cardiovascular or cerebrovascular diseases, cancer, immune deficiency and aging [1,2]. Saponins, commonly known as ginsenosides, are the main active ingredients in *Panax ginseng*. Among more than 150 ginsenosides identified currently [3], ginsenoside Rd (Rd) has been attracting more and more attention.

**Figure 1:**
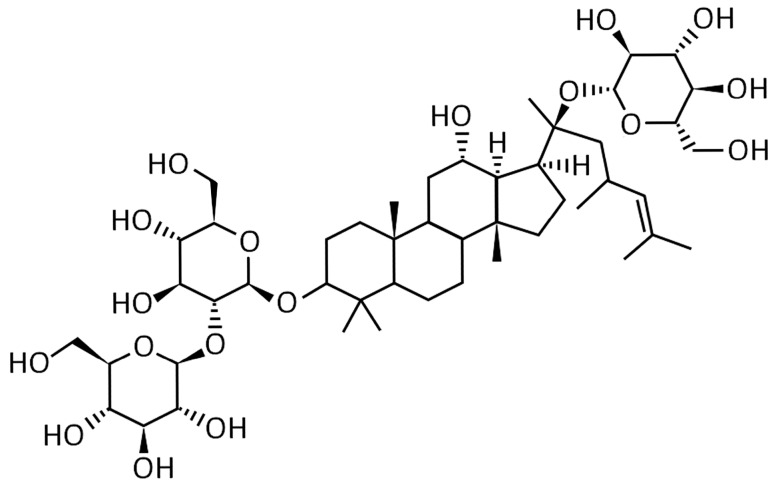
Chemical structure of Rd: Hydroxydammar-24-en-3-yl-2-*O*-β-d-glucopyranosyl-β-d-glucopyranoside.

Rd ((3β,12β)-20-(β-d-Glucopyranosyloxy)-12-hydroxydammar-24-en-3-yl-2-*O*-β-d-glucopyranosyl-β-d-glucopyranoside, Figure 1) is one of the major ginsenosides in the ginseng root. It has been reported that Rd could exert a remarkable neuroprotective effect on cerebral ischemia. In the phases II and III multicenter clinical trials, Rd showed safety and efficacy for the treatment of acute ischemic stroke [4,5]. In rats subjected to middle cerebral artery occlusion injury, Rd improved neurological outcome, attenuated infarct volume, ameliorated mitochondrial dysfunction, reduced oxidative damage, promoted glutamate clearance, and blocked apoptosis inducing factor mitochondrio-nuclear translocation and NF-κB nuclear accumulation [6,7,8,9,10,11]. In cultured neuronal cells subjected to hydrogen peroxide- or oxygen-glucose deprivation-induced injury, Rd protected the cells by reducing the intracellular levels of reactive oxygen species, enhancing the antioxidant enzymatic activities, stabilizing the mitochondrial membrane potential, and inhibiting Ca^2+^ influx [12,13,14]. All the evidence above suggested that Rd may be a potential neuroprotectant with multiple advantages for the treatment of ischemic stroke.

Apart from neuronal survival, which is usually a concern in most studies, another crucial event during ischemic stroke is axonal outgrowth or regeneration. It has been reported that axonal outgrowth and the formation of new connections are causally associated with functional recovery [15,16,17,18]. Considering the distinctive neuroprotective effects of Rd against ischemic stroke, we asked whether Rd could have similar beneficial effects on axonal outgrowth of neuronal cells. Therefore, in the present study, we utilized PC12 cells, a widely-used neuronal cell line, to examine the possible effects of Rd on their neurite outgrowth, and underlying molecular mechanisms were further explored as well.

## 2. Results

### 2.1. Rd Promotes Neurite Outgrowth of PC12 Cells

**Figure 2:**
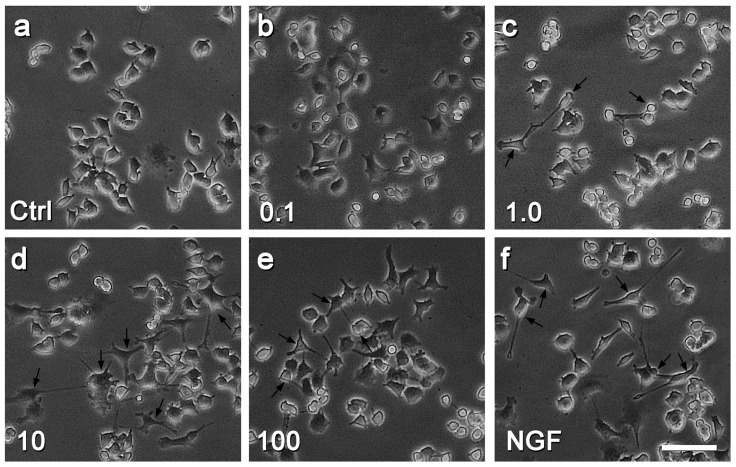
Effects of Rd on the neurite outgrowth of PC12 cells. Cell morphology is shown after PC12 cells were treated with saline (Ctrl, **a**), various concentrations (0.1–100 μM) of Rd (**b**–**e**), or 50 ng/mL nerve growth factor (NGF) (**f**) for three days. Arrows indicate the neurite-bearing cells in different groups. Scale bar: 50 μm.

**Figure 3:**
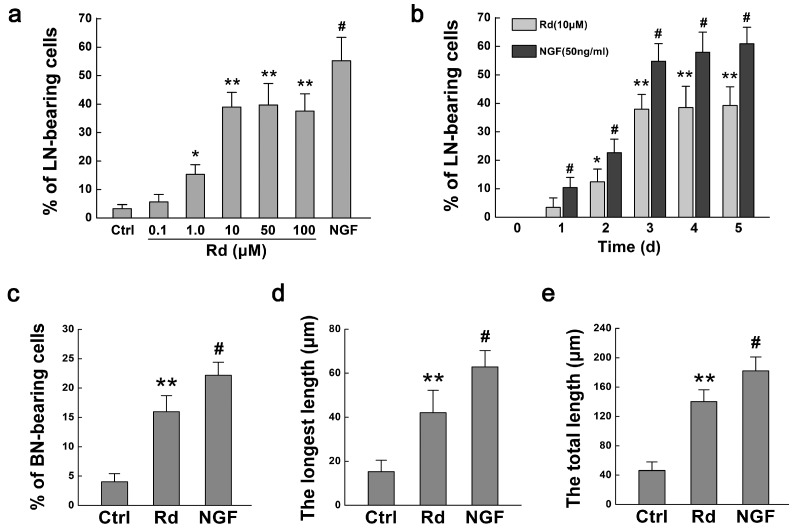
Quantitation of Rd-induced neurite outgrowth of PC12 cells. (**a**,**b**) The changes in the percentages of long neurite (LN)-bearing cells when PC12 cells were treated with various concentrations (0.1–100 μM) of Rd at 3 days (**a**), and with 10 μM Rd at different culturing time (0–5 days, **b**); (**c**) The changes in the percentages of branching neurite (BN)-bearing cells when PC12 cells were treated with 10 μM Rd at 3 days; (**d**,**e**) The changes in the length of longest neurites (**d**) and the total length of neurites (**e**) in PC12 cells treated with 10 μM Rd or 50 ng/mL NGF at 3 days. * *p* < 0.05; ** *p* < 0.01 (*vs.* the control group); # *p* < 0.01 (*vs.* respective Rd group).

Firstly we evaluated the effects of Rd on neurite outgrowth of PC12 cells by observing the cell morphology, counting the numbers of long neurite (LN)- and branching neurite (BN)-bearing cells, and measuring the length of neurites as described in the Materials and Methods section. When PC12 cells were treated with different concentrations of Rd or 50 ng/mL nerve growth factor (NGF) for three days, compared with the control (Figure 2a), more and longer neurites were observed (black arrows, Figure 2b–f). Rd at the concentrations of 1.0 μM (15.34% ± 3.41%, *p* < 0.05), 10 μM (38.95% ± 5.22%, *p* < 0.01), 50 μM (39.74% ± 7.56%, *p* < 0.01), or 100 μM (37.57% ± 6.08%, *p* < 0.01) but not 0.1 μM (5.66% ± 2.65%, *p* > 0.05), significantly increased the percentages of LN-bearing cells, compared with the control (3.31% ± 1.42%) (Figure 3a). It was noted that 10 μM of Rd had similar effects to that of 50 and 100 μM but less than that of NGF (55.25% ± 8.22%, *p* < 0.05). Additionally, we found that both Rd (10 μM) and NGF exerted their obvious promotive effects on neurite outgrowth after three days of treatment (Figure 3b). Thus, 100 μM and three-day time point were used in the following studies.

Next, we examined the changes in branching neurite (BN)-bearing cells as well. Our results showed that Rd increased the percentage of BN-bearing cells significantly (15.94% ± 2.75%, *p* < 0.01), compared with the control (4.02% ± 1.41%), though less than NGF (22.16% ± 2.22%, *p* < 0.05) (Figure 3c). Moreover, we measured the absolute length of neurites of PC12 cells. The results showed that the length of longest neurite in Rd-treated cells (42.03% ± 10.25 μm, *p* < 0.01) was longer than that of the control (15.23 ± 5.27 μm), though still shorter than that of NGF-treated cells (62.90 ± 7.42 μm, *p* < 0.05) (Figure 3d). Consistently, similar results were observed in the total length of neurites in Rd-treated cells (140.10 ± 16.25 μm, *p* < 0.01) as compared with the control (46.25 ± 11.76 μm, Figure 3e). All these data above indicated that Rd could promote the neurite outgrowth of PC12 cells.

### 2.2. Rd Activates MAPK/ERK and PI3K/AKT but Not PKC Signaling Pathways

**Figure 4:**
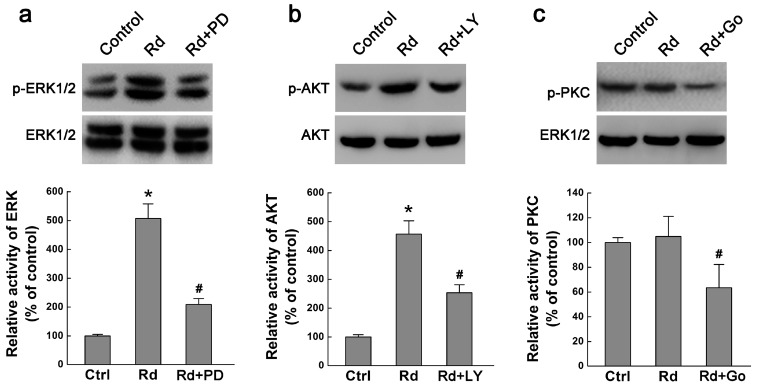
Effects of Rd on ERK, AKT and PKC phosphorylation. Western blot showed the changes in the phosphorylation levels of ERK1/2 (p-ERK1/2, **a**), AKT (p-AKT, **b**), PKC (p-PKC, **c**) in PC12 cells. Total protein levels of ERK1/2, AKT, and PKC were used as respective internal controls. PD, PD98059; LY, LY294002; Go, Go6976. * *p* < 0.05 (*vs.* the control group); # *p* < 0.01 (*vs.* the Rd group).

We next explored the possible mechanisms underlying the promotive effect of Rd on neurite outgrowth. We focused on MAPK/ERK, PI3K/AKT, and PKC signaling pathways, three well-established pathways involved in neurite outgrowth [19,20,21,22]. Since phosphorylation state is the activated form of ERK1/2, AKT, and PKC, we examined the effects of Rd on the phosphorylation levels of these kinases by Western blotting assays. Our results showed that Rd elevated the phosphorylation levels of ERK1/2 (p-ERK1/2) and AKT (p-AKT) but not PKC (p-PKC) in PC12 cells. Importantly, MAPK/ERK1/2 signaling inhibitor PD98059 (10 μM) and PI3K/AKT signaling inhibitor LY294002 (10 μM) could attenuate Rd-induced increased levels of p-ERK1/2 and *p*-AKT (Figure 4a,b). However, Rd seemingly did not affect the level of p-PKC, though PKC inhibitor Go6976 (3 μM) can mitigate PKC phosphorylation (Figure 4c). It was noted that Rd did not affect the total protein expression of either ERK1/2, AKT or PKC (Figure 4a–c). These results indicate that Rd may activate MAPK/ERK- and PI3K/AKT- but not PKC-mediated signaling pathways to exert its effects.

### 2.3. ERK and ARK Are Involved in Rd-Induced Neurite Outgrowth of PC12 Cells

**Figure 5:**
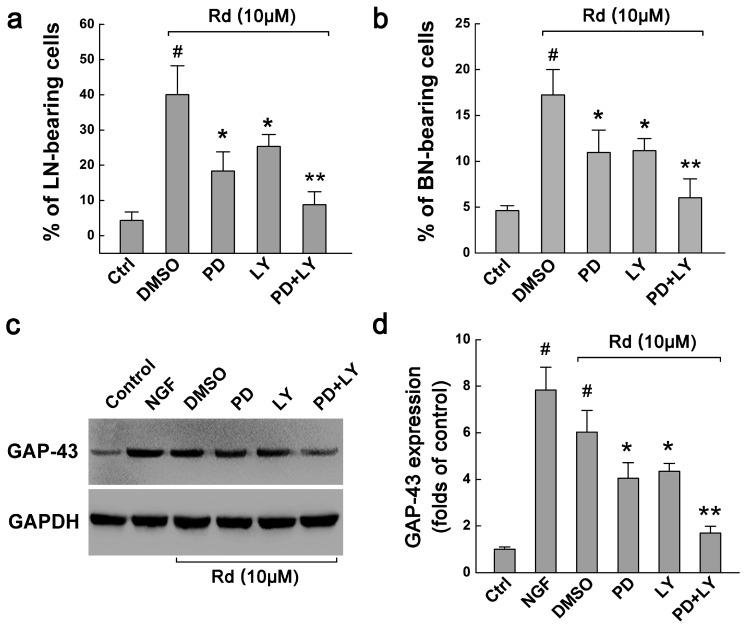
Involvement of ERK and AKT signals in Rd-induced neurite outgrowth. (**a**,**b**) The effects of ERK inhibitor PD98059 (PD) and AKT inhibitor LY294002 (LY) on the percentages of LN-(**a**) and BN-bearing cells (**b**) when PC12 cells were treated with 10 μM Rd at 3 days; (**c**,**d**) Western blot showed the effects of PD98059 and LY294002 on the expression of GAP-43 in PC12 cells treated with 10 μM Rd at 3 days. GAPDH was used as the internal control. # *p* < 0.01 (*vs.* the control group); * *p* < 0.05, ** *p* < 0.01 (*vs.* the DMSO group).

To further determine whether ERK1/2 or AKT activation may account for Rd-induced neurite outgrowth, we investigated the involvement of ERK and AKT signalings on neurite outgrowth. Our results showed that in Rd-treated PC12 cells, PD98059 and LY294002 decreased the percentages of LN-bearing cells from original 40.05% ± 8.22% to 18.34% ± 5.47% (*p* < 0.05) and 25.34% ± 3.41% (*p* < 0.05), respectively. When PD98059 and LY294002 were applied together, they further decreased the percentage of LN-bearing cells to 8.78% ± 3.69% (*p* < 0.01, Figure 5a). Similar results were also observed in the cells with BN, namely, PD98059, LY294002, and combination of the two attenuated the percentages of BN-bearing cells from original 17.24% ± 2.75% to 10.96% ± 2.45% (*p* < 0.05), 11.16% ± 1.32% (*p* < 0.05) and 6.02% ± 2.06% (*p* < 0.01), respectively (Figure 5b). Therefore, these results indicated that ERK and ARK signaling pathways could be involved in Rd-induced neurite outgrowth of PC12 cells.

### 2.4. Rd Upregulates ERK- and AKT-Mediated GAP-43 Expression to Promote Neurite Outgrowth

MAPK/ERK and PI3K/AKT pathways have been shown to be necessary for the expression of GAP-43 (growth-associated protein of 43 kDa), a neuron-specific protein associated with axon growth and growth cone formation that also regulates neurite outgrowth [23,24]. Therefore, we finally investigated whether Rd could affect the expression of GAP-43 via MAPK/ERK and PI3K/AKT signaling pathways. Western blotting results showed that in the control cells, only weak expression of GAP-43 was present. When NGF and Rd were applied, GAP-43 expression was significantly elevated, 7.82 ± 1.00 (*p* < 0.01) and 6.03 ± 0.90 folds (*p* < 0.01) higher than the control, respectively (Figure 5c,d). Moreover, PD98059, LY294002, and combination of the two decreased Rd-induced increased expression of GAP-43 by 32.90% (*p* < 0.05), 27.98% (*p* < 0.05) and 71.96% (*p* < 0.01), respectively. These results suggested that Rd may promote the neurite outgrowth of PC12 cells by upregulating GAP-43 expression via MAPK/ERK- and PI3K/AKT-dependent pathways.

## 3. Discussion

A series of studies have demonstrated that Rd showed profound neuroprotective effects with multiple advantages [6,7,8,9,10,11,12,13,14]. In the present study, we showed that Rd also promoted neuronal cells’ neurite outgrowth, a crucial process associated with functional recovery during neuronal repair [18]. To be more specific, Rd not only increased the numbers of LN- and BN-bearing cells, but also elongated the longest neurites and increased the total length of neurites in PC12 cells (Figure 2 and Figure 3), though its neurotrophic effects were less than that of NGF. These data suggest that in addition to the action on neuronal cells themselves, Rd may also exert its neuroprotective effects by promoting neurite outgrowth.

A variety of molecules or signaling pathways were reported to be involved in neurite outgrowth or axonal regeneration [19,20,21,22,25,26,27]. Among these, MAPK/ERK, PI3K/AKT, and PKC pathways are most frequently studied. For instance, it was reported that MEK/ERK or PI3K/AKT signaling activation was involved in TRPC6 channel-mediated neurite outgrowth in PC12 cells and hippocampal neurons [28], in brimonidine-mediated axon growth after optic nerve injury [29], in BIG1-regulated neurite development [30], in puerarin-regulated neuritogenesis in the neurite extension process [31], and in a natural diarylheptanoid-promoted neuronal differentiation and neurite outgrowth *in vitro* and *in vivo* [32]. On the other hand, PKC was found to be expressed in the optic pathway, and involved in axon routing at the optic chiasm [33], and in Orexin A-induced neurite outgrowth in primary cultured cortical neurons [34]. On the contrary, PKC inhibition blocked Wnt3a-dependent neurite formation in TC-32 cells [35]. All these data suggested that neurite or axon outgrowth or regeneration required the involvement of ERK, AKT, or PKC signals. Since previous studies have demonstrated that Rd can exert its protective effects via activating ERK and AKT pathways [11,36,37,38,39], we examined whether ERK or AKT was responsible for Rd-induced neurite outgrowth in PC12 cells as well. As expected, our results showed that Rd could activate the ERK and AKT signaling pathways, and application of their respective antagonists PD98059 and LY294002 partially inhibited Rd-elicited neurite outgrowth (Figure 4 and Figure 5). Importantly, when PD98059 and LY294002 were administered together, Rd’s action on neurites was almost completely blocked, implicating that Rd exerted its effects mainly via ERK and AKT signals. By contrast, our results revealed that the PKC pathway may have no significant involvement in Rd-induced neurite outgrowth.

It has been reported that the MAPK/ERK and PI3K/AKT pathways could regulate the expression of GAP-43, a neuron-specific protein involved in axonal outgrowth and regeneration [23,24]. GAP-43 overexpression strikingly potentiated the action of NGF on neurite initiation and regeneration in PC12 cells [40] while GAP-43 inhibition prevented the neuritogenesis of neuroblastoma cells [41]. The present study showed that Rd increased the expression of GAP-43 significantly, which could be partially attenuated by either ERK inhibitor PD98059 or AKT inhibitor LY294002, and almost completely blocked by the combination of the two (Figure 5), implying that Rd may regulate GAP-43 expression via ERK and AKT pathways. 

## 4. Materials and Methods 

### 4.1. Materials

Ginsenoside Rd was purchased from Tai-He Biopharmaceutical Co., Ltd. (Guangzhou, China), and prepared in the saline containing 10% propanediol. Poly-l-lysine hydrobromide was purchased from Sigma-Aldrich Co. (St. Louis, MO, USA). PI3K/AKT kinase inhibitor LY294002, MAPK/ERK1/2 kinase inhibitor PD98059, and PKC kinase inhibitor Go6976 were from Promega (Madison, WI, USA), and prepared in the saline containing 0.1% DMSO (Sigma-Aldrich). Human recombinant NGF was from Alomone Labs (Jerusalem, Israel). Anti-GAP-43 antibody was from Abcam (Cambridge, UK). Anti-p44/p42 MAPK (ERK1/2), anti-phospho-p44/p42 MAPK (ERK1/2) (Thr202/Tyr204), anti-Akt (pan), anti-phospho-Akt (Ser473), anti-PKC, and anti-phospho-PKC (pan, gamma Thr514) antibodies were purchased from Cell Signaling Technology (Beverly, MA, USA).

### 4.2. Cell Culture

PC12 cells (Health Science Research Resources Bank, Osaka, Japan) were seeded in 25 cm^2^ tissue culture flasks (Nunc, Roskilde, Denmark) and cultured in Dulbecco Eagle’s minimum essential medium (DMEM, Gibco-BRL, Grand Island, NY, USA) supplemented with 10% heat-inactivated horse serum (Invitrogen, Carlsbad, CA, USA) and 5% fetal bovine serum (FBS, Gibco-BRL, Grand Island, NY, USA), 100 U/mL penicillin, and 100 U/mL streptomycin in a humidified atmosphere containing 5% CO_2_ at 37 °C. 

### 4.3. Grouping

PC12 cells were suspended in the complete medium, and then plated on plastic culture dishes (Nunc, Denmark) coated with poly-l-lysine (30–70 kDa). After 24 h of culture, the medium was changed to that with 1% horse serum and 0.5% FBS, and then the indicated drugs were administered, thus establishing three groups: (1) the NGF group, containing 50 ng/mL NGF in the medium; (2) the Rd group, containing different concentrations (0.1, 1, 10, 50 or 100 μM) of Rd in the medium; (3) the control group: neither Rd nor NGF being added in the medium. Each group included at least 6 samples. Medium containing Rd or NGF was replaced every day. After 72 h, the cells were ready for the following experiments.

### 4.4. Evaluation of Neurite Outgrowth

For analysis of neurite outgrowth, PC12 cells were plated in 24-well plastic plates coated with poly-l-lysine at a density of 5 × 10^4^ cells/mL with indicated reagents for the indicated periods. Neurite outgrowth was evaluated by the percentage of neurite-bearing cells and the absolute length of cell neurite. Morphological changes of PC12 cells were observed under an IX70 microscope at different culturing time. The classification of neurite-bearing cells and their numbers were determined according to a previous report of Huffaker *et al.* (1984) [42]. In brief, in five random fields from each sample at each time points at 200×, the cells were defined as long neurite (LN)-bearing cells when their neurites were >2 cell-body in length, and as branching neurite (BN)-bearing cells when their neurites possessed at least one branch (≥5 μm). After counting the total number of neurite-bearing cells, the percentages of LN- or BN-bearing cells were calculated. For quantitation of the absolute length, the cultures were first subjected to HE staining, and then cell neurites were measured under an image analysis system (Leica Q570C, Heidelberg, Germany) at 200×. Random five fields from each example were measured. The length of the longest neurites (the longest neurite of a cell), the total length of neurites (the sum of all neurites’ length of a cell) and their means were calculated.

### 4.5. Western Blot

PC12 cells were plated in 6-well plastic plates coated with poly-l-lysine at a density of 1 × 10^6^ cells/mL with indicated reagents for the indicated periods. The cultured cells were collected, and total proteins were extracted using RIPA lysis buffer (PBS, 1% NP40, 0.5% sodium deoxycholate, 0.1% SDS, 0.25 mm PMSF, 5 mg/mL aprotinin, 1 mm sodium orthovanadate). After electrophoresed on 10% SDS-polyacrylamide gels, proteins were transferred onto nitrocellulose membranes, which were incubated at 4 °C overnight with following antibodies: anti-GAPDH, anti-GAP-43, anti-ERK1/2, anti-phospho-ERK1/2, anti-Akt, anti-phospho-Akt, anti-PKC, and anti-phospho-PKC. GAPDH was used as internal controls. After three washes, the blots were incubated with horseradish peroxidase-conjugated secondary antibodies (Cell Signaling Technology) for 1 h, and then the proteins were detected by an ECL chemiluminescence system (Amersham Biosciences, Piscataway, NJ, USA). All band signals were quantified using Image-J 1.42 (NIH Image software, available online: http://rsb.info.nih.gov/nih-image) and the data acquired were normalized to internal control expression and further normalized to the controls.

### 4.6. Statistical Analysis

All experiments above were repeated three times at least. The data were expressed as the mean ± SD and analyzed statistically with one-way analysis of variance (ANOVA) followed by a Turkey test using SPSS 12.0 software. *p*-values <0.05 were considered to be statistically significant.

## 5. Conclusions

In summary, here we provided the first evidence that Rd could promote neurite outgrowth in PC12 cells. This effect may be due to its ability to upregulate GAP-43 expression via MAPK/ERK- and PI3K/AKT-dependent pathways. Our findings may explain the mechanisms of the beneficial effects of Rd in the treatment of ischemic stroke. Admittedly, further studies using appropriate animal models are needed to explore the full potential of Rd in the treatment of ischemic CNS injuries.

## References

[1] Chen C.F., Chiou W.F., Zhang J.T. (2008). Comparison of the pharmacological effects of *Panax ginseng* and *Panax quinquefolium*. Acta Pharmacol. Sin..

[2] Ng T.B. (2006). Pharmacological activity of sanchi ginseng (*Panax notoginseng*). J. Pharm. Pharmacol..

[3] Christensen L.P. (2009). Ginsenosides chemistry, biosynthesis, analysis, and potential health effects. Adv. Food Nutr. Res..

[4] Liu X., Wang L., Wen A., Yang J., Yan Y., Song Y., Ren H., Wu Y., Li Z., Chen W. (2012). Ginsenoside-Rd improves outcome of acute ischaemic stroke—A randomized, double-blind, placebo-controlled, multicenter trial. Eur. J. Neurol..

[5] Liu X., Xia J., Wang L., Song Y., Yang J., Yan Y., Ren H., Zhao G. (2009). Efficacy and safety of ginsenoside-Rd for acute ischaemic stroke: A randomized, double-blind, placebo-controlled, phase II multicenter trial. Eur. J. Neurol..

[6] Ye R., Yang Q., Kong X., Han J., Zhang X., Zhang Y., Li P., Liu J., Shi M., Xiong L. (2011). Ginsenoside Rd attenuates early oxidative damage and sequential inflammatory response after transient focal ischemia in rats. Neurochem. Int..

[7] Ye R., Zhang X., Kong X., Han J., Yang Q., Zhang Y., Chen Y., Li P., Liu J., Shi M. (2011). Ginsenoside Rd attenuates mitochondrial dysfunction and sequential apoptosis after transient focal ischemia. Neuroscience.

[8] Ye R., Kong X., Yang Q., Zhang Y., Han J., Zhao G. (2011). Ginsenoside Rd attenuates redox imbalance and improves stroke outcome after focal cerebral ischemia in aged mice. Neuropharmacology.

[9] Ye R., Kong X., Yang Q., Zhang Y., Han J., Li P., Xiong L., Zhao G. (2011). Ginsenoside Rd in experimental stroke: Superior neuroprotective efficacy with a wide therapeutic window. Neurotherapeutics.

[10] Hu G., Wu Z., Yang F., Zhao H., Liu X., Deng Y., Shi M., Zhao G. (2013). Ginsenoside Rd blocks AIF mitochondrio-nuclear translocation and NF-κB nuclear accumulation by inhibiting poly(ADP-ribose) polymerase-1 after focal cerebral ischemia in rats. Neurol. Sci..

[11] Zhang X., Shi M., Bjoras M., Wang W., Zhang G., Han J., Liu Z., Zhang Y., Wang B., Chen J. (2013). Ginsenoside Rd promotes glutamate clearance by up-regulating glial glutamate transporter GLT-1 via PI3K/AKT and ERK1/2 pathways. Front. Pharmacol..

[12] Ye R., Han J., Kong X., Zhao L., Cao R., Rao Z., Zhao G. (2008). Protective effects of ginsenoside Rd on PC12 cells against hydrogen peroxide. Biol. Pharm. Bull..

[13] Ye R., Li N., Han J., Kong X., Cao R., Rao Z., Zhao G. (2009). Neuroprotective effects of ginsenoside Rd against oxygen-glucose deprivation in cultured hippocampal neurons. Neurosci. Res..

[14] Zhang C., Du F., Shi M., Ye R., Cheng H., Han J., Ma L., Cao R., Rao Z., Zhao G. (2012). Ginsenoside Rd protects neurons against glutamate-induced excitotoxicity by inhibiting Ca^2+^ influx. Cell. Mol. Neurobiol..

[15] Brown C.E., Aminoltejari K., Erb H., Winship I.R., Murphy T.H. (2009). *In vivo* voltage-sensitive dye imaging in adult mice reveals that somatosensory maps lost to stroke are replaced over weeks by new structural and functional circuits with prolonged modes of activation within both the peri-infarct zone and distant sites. J. Neurosci..

[16] Dancause N., Barbay S., Frost S.B., Plautz E.J., Chen D., Zoubina E.V., Stowe A.M., Nudo R.J. (2005). Extensive cortical rewiring after brain injury. J. Neurosci..

[17] Li S., Overman J.J., Katsman D., Kozlov S.V., Donnelly C.J., Twiss J.L., Giger R.J., Coppola G., Geschwind D.H., Carmichael S.T. (2010). An age-related sprouting transcriptome provides molecular control of axonal sprouting after stroke. Nat. Neurosci..

[18] Overman J.J., Clarkson A.N., Wanner I.B., Overman W.T., Eckstein I., Maguire J.L., Dinov I.D., Toga A.W., Carmichael S.T. (2012). A role for ephrin-A5 in axonal sprouting, recovery, and activity-dependent plasticity after stroke. Proc. Natl. Acad. Sci. USA..

[19] Bouquet C., Nothias F. (2007). Molecular mechanisms of axonal growth. Adv. Exp. Med. Biol..

[20] Schuldiner O., Yaron A. (2015). Mechanisms of developmental neurite pruning. Cell. Mol. Life Sci..

[21] Teng F.Y., Tang B.L. (2006). Axonal regeneration in adult CNS neurons-signaling molecules and pathways. J. Neurochem..

[22] Wood M.D., Mackinnon S.E. (2015). Pathways regulating modality-specific axonal regeneration in peripheral nerve. Exp. Neurol..

[23] Benowitz L.I., Routtenberg A. (1997). GAP-43: An intrinsic determinant of neuronal development and plasticity. Trends Neurosci..

[24] Korshunova I., Mosevitsky M. (2010). Role of the growth-associated protein GAP-43 in NCAM-mediated neurite outgrowth. Adv. Exp. Med. Biol..

[25] Cui Q. (2006). Actions of neurotrophic factors and their signaling pathways in neuronal survival and axonal regeneration. Mol. Neurobiol..

[26] Hinman J.D. (2014). The back and forth of axonal injury and repair after stroke. Curr. Opin. Neurol..

[27] Raivich G., Makwana M. (2007). The making of successful axonal regeneration: Genes, molecules and signal transduction pathways. Brain Res. Rev..

[28] Heiser J.H., Schuwald A.M., Sillani G., Ye L., Muller W.E., Leuner K. (2013). TRPC6 channel-mediated neurite outgrowth in PC12 cells and hippocampal neurons involves activation of RAS/MEK/ERK, PI3K, and CAMKIV signaling. J. Neurochem..

[29] Fujita Y., Sato A., Yamashita T. (2013). Brimonidine promotes axon growth after optic nerve injury through ERK phosphorylation. Cell Death Dis..

[30] Zhou C., Li C., Li D., Wang Y., Shao W., You Y., Peng J., Zhang X., Lu L., Shen X. (2013). BIG1, a brefeldin A-inhibited guanine nucleotide-exchange protein regulates neurite development via PI3K-AKT and ERK signaling pathways. Neuroscience.

[31] Zhao J., Cheng Y.Y., Fan W., Yang C.B., Ye S.F., Cui W., Wei W., Lao L.X., Cai J., Han Y.F. (2015). Botanical drug puerarin coordinates with nerve growth factor in the regulation of neuronal survival and neuritogenesis via activating ERK1/2 and PI3K/AKT signaling pathways in the neurite extension process. CNS Neurosci. Ther..

[32] Tang G., Dong X., Huang X., Huang X.J., Liu H., Wang Y., Ye W.C., Shi L. (2015). A natural diarylheptanoid promotes neuronal differentiation via activating ERK and PI3K-AKT dependent pathways. Neuroscience.

[33] Wang L., Lam J.S., Zhao H., Wang J., Chan S.O. (2014). Localization of protein kinase c isoforms in the optic pathway of mouse embryos and their role in axon routing at the optic chiasm. Brain Res..

[34] Bjornstrom K., Turina D., Strid T., Sundqvist T., Eintrei C. (2014). Orexin A inhibits propofol-induced neurite retraction by a phospholipase D/protein kinase Cε-dependent mechanism in neurons. PLoS ONE.

[35] Greer Y.E., Fields A.P., Brown A.M., Rubin J.S. (2013). Atypical protein kinase Cι is required for Wnt3a-dependent neurite outgrowth and binds to phosphorylated Dishevelled 2. J. Biol. Chem..

[36] Tamura T., Cui X., Sakaguchi N., Akashi M. (2008). Ginsenoside Rd prevents and rescues rat intestinal epithelial cells from irradiation-induced apoptosis. Food Chem. Toxicol..

[37] Wang Y., Li X., Wang X., Lau W., Wang Y., Xing Y., Zhang X., Ma X., Gao F. (2013). Ginsenoside Rd attenuates myocardial ischemia/reperfusion injury via AKT/GSK-3β signaling and inhibition of the mitochondria-dependent apoptotic pathway. PLoS ONE.

[38] Zhang X., Shi M., Ye R., Wang W., Liu X., Zhang G., Han J., Zhang Y., Wang B., Zhao J. (2014). Ginsenoside Rd attenuates Tau protein phosphorylation via the PI3K/AKT/GSK-3β pathway after transient forebrain ischemia. Neurochem. Res..

[39] Zhou J.S., Wang J.F., He B.R., Cui Y.S., Fang X.Y., Ni J.L., Chen J., Wang K.Z. (2014). Ginsenoside Rd attenuates mitochondrial permeability transition and cytochrome *c* release in isolated spinal cord mitochondria: Involvement of kinase-mediated pathways. Int. J. Mol. Sci..

[40] Yankner B.A., Benowitz L.I., Villa-Komaroff L., Neve R.L. (1990). Transfection of PC12 cells with the human GAP-43 gene: Effects on neurite outgrowth and regeneration. Brain Res. Mol. Brain Res..

[41] Shea T.B., Perrone-Bizzozero N.I., Beermann M.L., Benowitz L.I. (1991). Phospholipid-mediated delivery of anti-GAP-43 antibodies into neuroblastoma cells prevents neuritogenesis. J. Neurosci..

[42] Huffaker T., Corcoran T., Wagner J.A. (1984). Adenosine inhibits cell division and promotes neurite extension in PC12 cells. J. Cell. Physiol..

